# Phylogenetic Analysis of the Complete Mitochondrial Genome of *Madurella mycetomatis* Confirms Its Taxonomic Position within the Order Sordariales

**DOI:** 10.1371/journal.pone.0038654

**Published:** 2012-06-06

**Authors:** Wendy W. J. van de Sande

**Affiliations:** Department of Medical Microbiology and Infectious Diseases, Erasmus MC, Rotterdam, The Netherlands; New York State Health Department and University at Albany, United States of America

## Abstract

**Background:**

*Madurella mycetomatis* is the most common cause of human eumycetoma. The genus *Madurella* has been characterized by overall sterility on mycological media. Due to this sterility and the absence of other reliable morphological and ultrastructural characters, the taxonomic classification of *Madurella* has long been a challenge. Mitochondria are of monophyletic origin and mitochondrial genomes have been proven to be useful in phylogenetic analyses.

**Results:**

The first complete mitochondrial DNA genome of a mycetoma-causative agent was sequenced using 454 sequencing. The mitochondrial genome of *M. mycetomatis* is a circular DNA molecule with a size of 45,590 bp, encoding for the small and the large subunit rRNAs, 27 tRNAs, 11 genes encoding subunits of respiratory chain complexes, 2 ATP synthase subunits, 5 hypothetical proteins, 6 intronic proteins including the ribosomal protein *rps*3. In phylogenetic analyses using amino acid sequences of the proteins involved in respiratory chain complexes and the 2 ATP synthases it appeared that *M. mycetomatis* clustered together with members of the order Sordariales and that it was most closely related to *Chaetomium thermophilum.* Analyses of the gene order showed that within the order Sordariales a similar gene order is found. Furthermore also the tRNA order seemed mostly conserved.

**Conclusion:**

Phylogenetic analyses of fungal mitochondrial genomes confirmed that *M. mycetomatis* belongs to the order of Sordariales and that it was most closely related to *Chaetomium thermophilum*, with which it also shared a comparable gene and tRNA order.

## Introduction

*Madurella mycetomatis* is the most common causative agent of human mycetoma, a chronic inflammatory disease, which remains localized and involves subcutaneous tissues, fascia and bones [1]. The disease is characterised by tumefaction, discharging sinuses and the presence of fungal grains [1]. The generic criteria for *Madurella* are primarily based on tissue morphology and overall sterility on mycological media, as well as an invasive potential in human and animal hosts [2]. Since no sexual stage of *M. mycetomatis* has been discovered, the taxonomic classification of *Madurella* has long been a challenge. Especially, since there are also no asexual conidia produced nor other morphological and ultractructural characters which could be of aid in the taxonomic classification. With the development of molecular typing tools, such as sequencing of the nuclear sequences encoding for the internal transcribed spacer (ITS), the beta-tubulin gene and the ribosomal binding protein it became possible to establish the taxonomic place of *Madurella* among the ascomycetes [3], [4]. Based on these nuclear sequence data, it appeared that the genus *Madurella*, consisting of five species *M. mycetomatis, M. grisea, M. pseudomycetomatis, M. fahalii* and *M. tropicana,* could be taxonomically differentiated into two different orders, namely the orders Sordariales and Pleosporales [3], [4]. The generic type species *M. mycetomatis* belonged to the order of Sordariales together with *M. pseudomycetomatis, M. fahalii* and *M. tropicana*, and the genus *Madurella* appeared to be closely related to the genus *Chaetomium* [3], [4].

Next to nuclear sequences it is also possible to use mitochondrial sequences for phylogenetic analyses. Mitochondria are considered descendants of an endosymbiotic α-proteobacterium that was engulfed by a eukaryotic or archeabacteria-like cell more than one billion years ago [5]. The current mitochondrial data points to a single origin of mitochondria with no transfer of mitochondria between different eukaryotes [6]. The mitochondrial DNA present in all mitochondria examined to date is believed to be a remnant of the original endosymbiont’s DNA, with the number of genes contained greatly reduced [5]. In filamentous fungi, the mitochondria are uniparental inherited and their genomes evolve faster than the corresponding nuclear DNA of the fungus [7], [8], [9]. Fungal mitochondrial genomes encode 5 to 100 genes, with a typical fungal mitochondrial core genome containing 14 conserved protein-coding genes, 2 rRNA genes and a variable number of tRNAs [5], [10]. MtDNA divergence between different fungal species is predominantly associated with variation in intergenic regions, intronic sequences and gene order, but the core protein-coding genes are conserved [11]. These core conserved protein-coding genes are convincing tools for phylogenetic analysis as they provide not only a large gene-set for which the sequences can be compared directly, but also the opportunity to compare the position of these genes [11]. With the development of novel sequence methods, the number of mitochondrial genomes of fungi has been expanded in the recent past [10], [11], [12], [13], [14], [15]. This gives us an opportunity to study the phylogeny of fungi using not only nuclear DNA but also mitochondrial DNA. Here we present the mitochondrial genome sequence of *M. mycetomatis.* Its gene order and amino-acid sequences are used in phylogenetic analyses to determine the place of *M. mycetomatis* in the fungal kingdom.

## Materials and Methods

### Isolate

*M. mycetomatis* strain mm55, isolated from the lesion of a 22-year-old patient seen in the Mycetoma Research Centre, University of Khartoum, Sudan, was used in this study. Written informed consent was obtained from this patient and ethical clearance was obtained from Soba University Hospital Ethical Committee. This strain was isolated by direct culture of the black grains obtained by a deep biopsy and identified by morphology, PCR-RFLP and sequencing of the ITS region [16]. This strain is used in the only mouse model of eumycetoma in use today and considered the type strain in phylogenetic and antifungal susceptibility testing as well [17], [18], [19], [20]. The strain was maintained on Sabouraud Dextrose Agar (Difco Laboratories, Paris, France) at 37°C. Passage to fresh medium was done on a monthly basis.

### DNA Extraction

Three-week-old *Madurella* cultures were scraped from Sabouraud agarplates, frozen in liquid nitrogen and ground with a mortar and pestle. DNA was extracted from the resulting pulp with the Promega Wizard Kit (Promega). To the grind mycelia, 300 µl lysis solution was added and mixed by pipetting gently. From this step onwards, the yeast protocol from the Promega Wizard Kit was used according to the manufacturer’s instructions.

### Sequencing and Assembling of the Mitochondrial Genome

The genome of *M. mycetomatis* was sequenced using Roche GS junior titanium 454 sequencing according to the manufactures instructions. In short DNA was fragmented by nebulisation to an average fragment length of 600–900 bp after which the fragments were amplified and coupled to capture beads using the emPCR amplification kit Lib-L for the GS Junior Titatium Series (roche). In total 5×10^6^ coupled beads were deposited on the GS junior titanium picotiterplates (Roche) and sequenced. To assemble the mitochondrial genome the GS de novo assembler of Roche was used. The two ends of the assembled sequence were amplified with primers mmmitofw (5′-TCATGGCTTAGATGTTGTGG-3′) and mmmitorv (5′-GAGCTATAGTGGCTCCTAGT-3′) and resequenced by sanger sequencing to confirm the circular nature of the mitochondrial genome.

### Annotation of the Mitochondrial Genome

Open reading frames (ORFs) were searched with CLC sequence viewer version 6.5.1 (CLC bio, Aarhus, Denmark) and annotated manually using the published mitochondrial genomes of *Podospora anserina, Sordaria macrospora* and *Neurospora crassa.* For hypothetical proteins a cut off of 100 amino acids was used. Codon usage was determined by using the Sequence Manipulation Suite version 2 (www.bioinformatics.org/sms2/codon_usage.html). tRNAs were identified by using tRNAscan-SE 1.21 [21], [22], ARAGORN v1.2 [23], ARWEN [24] and RNAweasel [25] software programs. A tRNA was determined to be a true tRNA if it was found with at least 2 out of 4 software programs.

### Phylogenetic Analysis

To compare the *M. mycetomatis* mitochondrial genome with the genome of other fungal mitochondrial genomes, the amino-acid sequences of the protein-encoding genes *atp6, atp8, atp9, cob, cox1, cox2, cox3, nad1, nad2, nad3, nad4, nad4L, nad5* and *nad6* were aligned by clustalW using the Mega 5.05 software package [26]. The sequences of the selected proteins were extracted from the fungal mitochondrial genomes deposited in the GenBank database. The aligned amino-acid sequences were used to construct a maximum likelihood tree with 1000 bootstrap replicates based on the cpREV model using Mega 5.05 [26].

### Genbank Accession Number

The mtDNA sequence of *M. mycetomatis* strain mm55 was deposited in GenBank under accession number JQ015302.

## Results and Discussion

### Genome Organization

**Figure 1:**
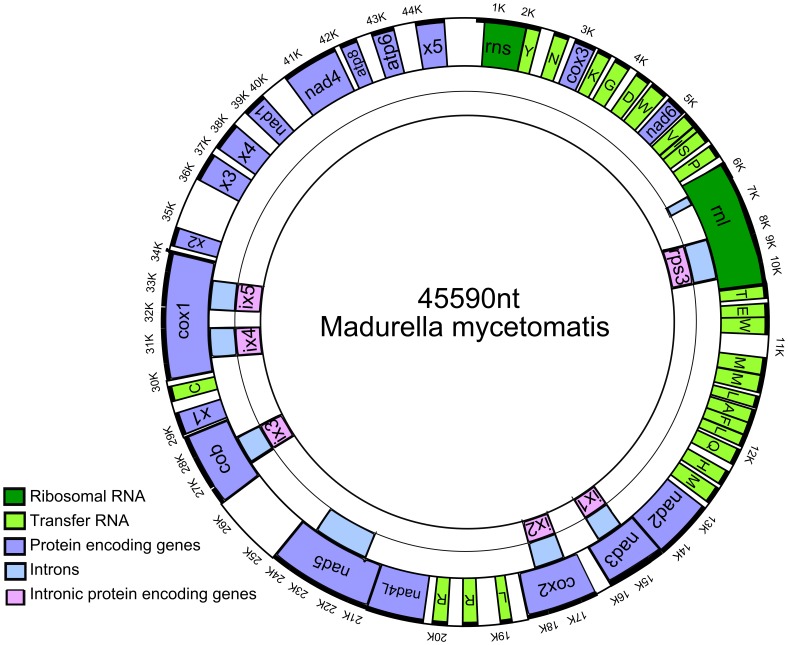
Physical map of the mitochondrial genome of *M. mycetomatis.* All genes are located on the plus-strand and are shown in the outer ring of the circle, they are transcribed counterclockwise. On the plus-strand the following genes are encountered: rnl, rns, 11 genes encoding subunits of respiratory chain complexes (*cob, cox1, cox2, cox3, nad1, nad2, nad3, nad4, nad4L, nad5,* and *nad6*), 2 ATP synthase subunits (*atp6* and *atp8*), 5 hypothetical proteins (x1, x2, x3, x4 and x5) and 6 intronic proteins including the ribosomal protein *rps3* are shown. The introns are shown as blue boxes in the middle ring underneath the genes in which they are located. The intronic proteins are shown as pink boxes in the inner ring underneath the introns and the genes in which they are located. The exact starting en ending positions of each gene, intron and tRNA are shown in table 1.

The mitochondrial genome of *M. mycetomatis* is a typical circular DNA molecule with a length of 45,590 bp. This mitochondrial genome size is small in comparison with the published mitochondrial genomes belonging to the order of the Sordariales. These genomes range from 64,840 nt (*Neurospora crassa* as stated by the Broad Institute)) to 127,206 nt *(Chaetomium thermophilum*) [14], [27], [28], [29]. This difference in genome size is due to the variation in intergenetic regions and the presence of hypothetical proteins. In overall, the mitochondrial genome of *M. mycetomatis* is highly compact, with 80% of the genome encoding for structural genes. The genome encodes for the small and the large subunit rRNAs, 27 tRNAs, 11 genes encoding subunits of respiratory chain complexes, 2 ATP synthase subunits, 5 hypothetical proteins and 6 intronic proteins including the ribosomal protein *rps3* (Figure 1, table 1). All genes and tRNAs are found on the plus-strand of the mitochondrial genome, as was also found for mitochondria of most other ascomycetes although for some ascomycetes such as *Mycosphaerella graminicola* genes were located both strands of the mitochondrial genome. [11], [13], [14], [27], [30], [31]. The *M. mycetomatis* mitochondrial genome is AT-rich, with an overall G+C content of only 26.8%. The regions of the encoding RNA genes, have a slightly higher G+C content, namely 28.8%. This is in agreement with values found for other fungal mitochondria [10].

**Table 1:** Genome organization of *M. mycetomatis.*

Gene							Intron			Intron protein						
Gene	Start position	Stop position	Length(nt)	Length (aa)	Start-Codon	Stop-codon	Intron Start	Intron Stop	Intron type	Gene	Start position	Stop position	Length (nt)	Length (aa)	Start-Codon	Stop-codon
rns	267	2050	1784													
*trn*Y	2042	2128	87													
*trn*N	2477	2547	71													
*cox*3	2721	3530	810	269	ATT	TAG										
*trn*K	3616	3687	72													
*trn*G	3825	3895	71													
*trn*D	4118	4190	73													
*trn*W	4283	4353	71													
*nad*6	4464	5129	666	221	ATG	TAA										
*trnV*	5201	5272	72													
*trn*I	5288	5359	72													
*trn*S	5364	5448	85													
*trn*P	5574	5648	75													
rnl	5823	10438	4616				6145	6271	IA							
							8269	9860	IA	*rps*3	8493	9740	1248	415	ATG	TAA
*trn*T	10459	10529	71													
*trn*E	10655	10735	81													
*trn*W	10655	10726	72													
*trn*M	11299	11371	73													
*trn*M	11386	11458	73													
*trn*L	11554	11638	85													
*trn*A	11697	11769	73													
*trn*F	11773	11845	73													
*trn*L	11856	11940	85													
*trn*Q	12029	12101	73													
*trn*H	12305	12377	73													
*trn*M	12482	12553	72													
*nad*2	12708	14390	1683	560	ATG	TAA										
*nad*3	14391	16094	1704	137	ATG	TAA	14480	15770	IC2	*ix*1	14391	15725	1335	444	ATG	TAA
*cox*2	16436	18502	2067	250	ATG	TAG	16640	17953	IC2	*ix*2	16643	17903	1260	419	ATG	TAA
*trn*L	18894	18967	74													
*trn*R	19515	19585	71													
*trn*R	19957	20029	73													
*nad*4L	20252	20521	270	89	ATG	TAA										
*nad*5	20521	24611	4091	643	ATG	TAA	21452	23604	IA							
*cob*	26392	28848	2457	395	ATG	TAA	26788	28053	ID	*ix*3	26761	27690	930	309	ATG	TAG
*x*1	28913	29464	552	183	ATG	TAG										
*trnC*	29762	29832	71													
*cox*1	29979	34122	4144	566	ATG	TAG	30367	31514	IB	*ix*4	30418	31407	990	329	ATG	TAA
							32189	33478	IB	*ix*5	32167	33465	1299	432	ATG	TAA
*x*2	34251	34715	465	154	ATG	TAA										
*x*3	34968	35975	1008	335	ATG	TAA										
x4	37164	38372	1209	402	ATT	TAA										
*nad*1	38765	39880	1116	371	ATG	TAA										
*nad*4	40576	42006	1431	476	ATG	TAA										
*atp*8	42159	42311	153	50	ATG	TAA										
*atp*6	42703	43491	789	262	ATG	TAA										
*x*5	44033	44881	849	282	ATG	TAA										

Genomic organization of the mitochondrial genome of *Madurella mycetomatis.* In this table the start and stop positions and the lengths of the resulting nucleotide and amino acid sequences are shown for of all genes, tRNAs, introns and intronic proteins. For the introns the group and subgroups are also stated. The classification of these introns are based on the conservation of the core sequences and structural motifs as indicated by Michel et al. [48].

### Protein Coding Genes

The *M. mycetomatis* mitochondrial genome has the following genes encoding proteins involved in respiratory chain complexes: ATP synthase subunits 6 and 8 (*atp6* and *atp8*), but not for subunit 9, apocytochrome b *(cob*), the cytochrome *c* oxidase subunits 1, 2, and 3 (*cox1, cox2* and *cox3)* and NADH dehydrognease subunits 1, 2, 3, 4, 4 L, 5, and 6 *(nad1, nad2, nad3, nad4L, nad5* and *nad6*) (table 1). Most of these proteins are highly conserved within fungal mitochondrial genomes [13], [27], [30], only for the *nad* genes and *atp*9 some variation is noted. No *nad* genes are present in most of the yeasts and in some fungi *atp*9 is located in the nuclear genome or on a different, independent circular molecule, rather than in the mitochondrial genome [12], [27], [32]. Next to the proteins involved in respiratory chain complexes, the mitochondrial DNA encodes for 5 hypothetical proteins and 6 intronic proteins including ribosomal protein S3 (*rps3*). Of the 5 hypothetical proteins only hypothetical proteins 1 and 3 do not show any homology with other known genes. For the other hypothetical genes some homology is found at the protein level. Hypothetical protein 2 shows homology with an unnamed protein product with accession number CAA38821, found in the mitochondrion of *Podospora anserina* (e-value: 3e-08, max identitiy 38%). Hypothetical protein 4 shows homology with YP_003127070, an GIY-YIG endonuclease found in an intronic protein in the *cob* gene of the yeast *Dekkera bruxellensis* (e-value: 5e-04, max identity 25%) [12]. Since no GIY-YIG motif is found in this hypothetical protein, it probably does not function as a GIY-YIG endonuclease. Hypothetical protein 5 shows homology with orf296 of *P. anserina* (Accesion number NP_074917, e-value:2e-19), UrfLM of *Neurospora intermedia* (Accession number AAU25928, e-value:3e-8) and an unnamed protein product of *N. crassa* (Accession number CAA31721.1, e-value: 5e-5). Orf 296 is in *P. anserina* a LAGLIDADG endonuclease found in an intronic sequence after exon3 of cox1 gene [27], [33]. Hypothetical protein 5 does not show a LAGLIDADG domain in its sequence and therefore probably does not function as a LAGLIDADG endonuclease. Hypothetical proteins 4 and 5 are probably remnants of endonucleases but do not function as endonucleases any more, there precise function, if any, remains unknown.

### Introns

In the coding genes of the mitochondrial genome of *M. mycetomatis,* a total of 8 introns are found. All introns are group I introns (table 1). Two introns are found in both the large ribosomal subunit (intron IA) and in *cox1* (both intron IB). Single introns are found in *cob* (intron ID), *cox2* (intron IC2), *nad3* (intron IC2) and *nad5* (intron IA) (table 1). Group I introns are considered to be mobile genetic elements which interrupt protein-coding and structural RNA genes [34]. One of the features of group I introns is that they themselves are often invaded with smaller genes that encode mobility-promoting activities that enables the DNA element to move within and between genomes, usually so-called homing endonucleases [34]. In the *M. mycetomatis* mitochondrial DNA we find five intronic proteins, located in the introns of *cob*, *cox1* (in each intron one), *cox2* and *nad*3 which encode for such homing endonucleases and one intronic protein which encode for ribosomal protein S3 (rps3). Of the four families of homing endonuclease proteins only endonucleases with the conserved amino acid sequence motifs LAGLIDADG (intron proteins 1 2, and 4) and GIY-YIG (intron proteins 3 and 5) are found. The endonuclease assignment was supported by BLAST analysis and motif identification using PFAM. LAGLIDADG homing endonucleases are found in two forms: a single LAGLIDADG motif that dimerizes and double-motif forms derived form a gene fusion event between two monomeric forms [35]. The endonucleases found in the *M. mycetomatis* mitochondrial genome are all with double-motif forms.

### Intergenic Regions

The presence of putative mitochondrial promoters are detected by comparison of the only promoter from the Sordariales, the *Neurospora crassa* sequence TTAG(A/T)RR(G/T)(G/C)N(A/T) [11], [36], [37]. Two putative promoter sequences are located within the intergenic regions and close to the 5′ end of coding genes, namely TTAGAATCTT (15885–15896) and TTAGTGGTCTA (36265–36276). Putative promoter sequence TTAGAATCTT is located 551 bp for the 5′ end of *cox*2, while putative promoter sequence TTAGAATCTT is located 899 bp for hypothetical protein 4. Both putative promoter sequences are preceded by a 15–23 bp long AT-rich region, as is also described for other fungal species, thus strengthening the hypothesis that these sequences may indeed be mitochondrial promoters [11], [37].

### Genetic Code and Codon Usage

Using the genetic mould mitrochondrial code from NCBI (translation table 4), the codon usage of the *M. mycetomatis* mitochondrial ORFs is determined. Of the 23 ORFs, only the *cox3* and hypothetical protein 3 starts with the ATT initiation codon, all other genes start with the ATG initiation codon (table 1). Most of the ORF end with the in preferred TAA stop-codon, only 5 ORFs (*cox*1, *cox*2, *cox*3, hypothetical protein 1 and intronprotein 3) end with the TAG stop-codon [38]. As is also found for other fungi, the most frequently used amino acid in the 23 protein genes is leucine followed by isoleucine (table 2) [11]. As seen in table 2, the codon usage in *M. mycetomatis* mitochondrial ORFs shows a strong bias towards codons ending with a U or A since 86.8% of the codons ends with these bases. The tendency for the A and U residues in the wobble position has also been observed in other fungal genomes [12], [39], [40], [41], [42]. As expected due to the high AU content of the mitochondrial genome, the preference of A and U residues is also noted in the overall codon use. The most frequently used codons consist only of Us and As and were UUA (9.04%), AUA (6.07%), AAU (5.68%), UUU (5.41%), AAA (5.17%), UAU (4.36%) and AUU (4.23%) (table 2). The least frequently used codons, CGC (0.02%), CGG (0.06%), CGG (0.06%), AGG (0.07%) and CCC (0.10%), are the codons which consist mainly of Cs and Gs (table 2).

**Table 2:** Codon usage in protein coding genes of *M. mycetomatis* mitrochondrial genome.

AA	codon	%	AA	codon	%	AA	codon	%	AA	Codon	%
A	GCG	0.22	H	CAU	1.31	P	CCG	0.09	S	UCU	3.11
A	GCA	1.46	H	CAC	0.36	P	CCA	0.64	S	UCC	0.26
A	GCU	2.43	I	AUA	6.07	P	CCU	2.48	T	ACG	0.15
A	GCC	0.26	I	AUU	4.23	P	CCC	0.10	T	ACA	2.11
C	UGU	0.61	I	AUC	0.56	Q	CAG	0.22	T	ACU	2.73
C	UGC	0.10	K	AAG	0.75	Q	CAA	1.90	T	ACC	0.16
D	GAU	2.92	K	AAA	5.17	R	AGG	0.07	V	GUG	0.44
D	GAC	0.45	L	UUG	0.85	R	AGA	2.23	V	GUA	2.78
E	GAG	0.82	L	UUA	9.04	R	CGG	0.06	V	GUU	2.16
E	GAA	3.38	L	CUG	0.26	R	CGA	0.11	V	GUC	0.17
F	UUU	5.41	L	CUA	1.01	R	CGU	0.42	W	UGG	0.14
F	UUC	1.44	L	CUU	1.46	R	CGC	0.02	W	UGA	1.11
G	GGG	0.50	L	CUC	0.11	S	AGU	3.31	Y	UAU	4.36
G	GGA	2.08	M	AUG	2.10	S	AGC	0.44	Y	UAC	0.75
G	GGU	3.22	N	AAU	5.68	S	UCG	0.15	Stop	UAG	0.06
G	GGC	0.11	N	AAC	0.84	S	UCA	1.79	Stop	UAA	0.23

The percentage codon used in the protein encoding regions of atp6, atp8, cob, cox1, cox2, cox3, nad1, nad2, nad3, nad4, nad4L, nad5, nad6, hypothetical protein 1, hypothetical protein 2, hypothetical protein 3, hypothetical protein 4, hypothetical protein 5, rps3, intron protein 1, intron protein 2, intron protein 3, intron protein 4 and intron protein 5 is depicted.

### tRNAs

In the *M. mycetomatis* mitochondrial genome 27 tRNAs are identified which clustered roughly in three groups (figure 1). Among the tRNAs all amino-acids are accounted for, but for some amino-acids multiple tRNAs are found (table 3). There are two tRNAs with different anticodons for arginine, four tRNAs with three different anticodons for leucine, three tRNAs with the same anticodon for methionine and two tRNAs with the same anticodon for tryptophane. All tRNAs have a cloverleaf structure except the tyrosine tRNA and the leucine tRNA with anticodon AAG, they have a TV-loop and D-loop structure respectively.

**Table 3:** tRNAs identified in the genome of *M. mycetomatis.*

AA	Anti-codon	AA	Anti-codon	AA	Anti-codon	AA	Anti-codon
A	UGC	I	GAU	P	UGG	W	UCA*
C	GCA	K	UUU	Q	UUC	Y	GUA
D	GUC	L	AAG	R	ACG		
E	UUG	L	UAA	R	UCU		
F	GAA	L	UAG*	S	UGA		
G	UCC	M	CAU**	T	UGU		
H	GUG	N	GUU	V	UAC		

* two tRNAs with the same anticodon were found.

** three tRNAs with the same anticodon were found.

### Phylogeny and Comparative Genomics

**Figure 2:**
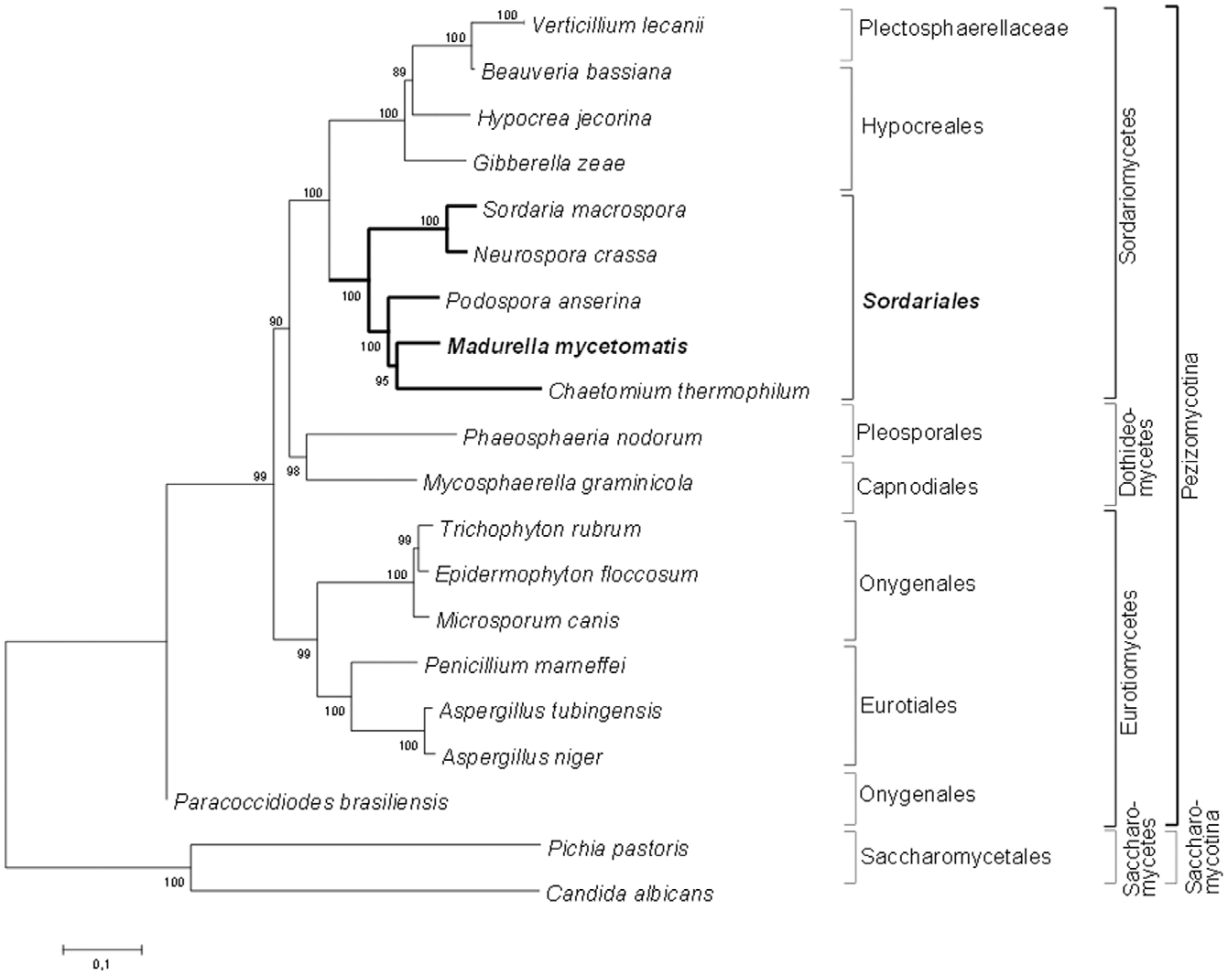
Maximum likelihood phylogenetic tree based on amino acid sequences of conserved mitochondrial proteins of various fungal species. Amino acid sequences of the genes *atp*6, *atp*8, *atp*9, *cob, cox*1, *cox*2, *cox*3, *nad*1, *nad*2, *nad*3, *nad*4, *nad*4L, *nad*5 and *nad*6 were used to construct this tree using the maximum likelihood algorithm of MEGA 5.05. Bootstrap support was calculated from 1000 replicates using the same program. GenBank sequences used were: *V. lecanni* (NC_004514), *B. bassiana* (NC_010652), *H. jecorina* (NC_NC003388), *G. zeae* (NC_009493), *S. macrospora* (CABT01004783), *P. anserina* (NC_001329), *C. thermophilum* (NC­_015893), *P. nodorum* (NC_009746), *T. rubrum* (NC_012824), *E. floccosum* (NC_007394), *M. canis* (NC_012832), *P. marneffei* (NC_005256), *A. tubingensis* (NC_007597), *A. niger* (NC_007445), *P. brasiliensis* (NC_007935), *P. pastoris* (NC_015384), *C. albicans* (NC_002653). Protein sequences of *N. crassa* mtDNA was downloaded from supercontig 10.21 from the Broad institute.

With the exception of the group of yeast that are lacking NADH genes, all other fungal mtDNAs contain the same essential functional genes [11]. Therefore, the sequences of these 14 conserved protein encoding genes, as well as the mitochondrial organization of these genes can be used tpone.0038654.g001.tifo determine the relations between different fungal species. Amino acid sequence of 14 protein coding genes in the mitochondrial genomes of *M. mycetomatis* and 20 other fungi are used for phylogenetic tree construction (figure 2). Most nodes in this tree have high bootstrap values which indicate the robustness of the tree computed. As found by others, the mitochondrial genomes of the yeast species cluster apart from the mitochondrial genomes obtained from filamentous fungi [10]. As is seen in figure 2, *M. mycetomatis* clusters amongst other species of the order Sordariales with high bootstrap support. Placing *M. mycetomatis* in the order Sordariales is in line with previous observations based on the nuclear sequences SSU, ITS, betatubulin 2 and ribosomal binding protein 2 [3], [4]. Based on an extensive phylogenetic comparison of the SSU rDNA sequence of *M. mycetomatis* with that of 157 other members of the Ascomycota belonging to the orders Chaetothyriales, Diaporthales, Dothideales, Eurotiales, Halosphaeriales, Hypocreales, Lecanorales, Leotiales, Microascales, Onygenales, Ophiostomatales, Pezizales, Pleosporales, Sordariales, Taphrinales and Tuberales it appeared that *M. mycetomatis* clustered among the members of the order Sordariales while *M. grisea* clustered among the members of the order Pleosporales [3]. In order to determine the phylogenetic place of *M. mycetomatis* within the order Sordariales, the ITS, betatubulin 2 and ribosomal binding protein 2 were also sequenced and compared to 39 members of the order Sordariales. In this latter study it appeared that *M. mycetomatis* was most closely related to *M. tropicana, M. pseudomycetomatis* and *M. fahalli,* but that the genus *Madurella* itself was most closely related to the genus *Chaetomium* [4]. This close relatedness to the genus *Chaetomium* is confirmed in this study. Based on the phylogenetic comparisons made with the mitochondrial sequence, it appears that the closest relative of *M. mycetomatis* is *C. thermophilum.*

**Figure 3:**
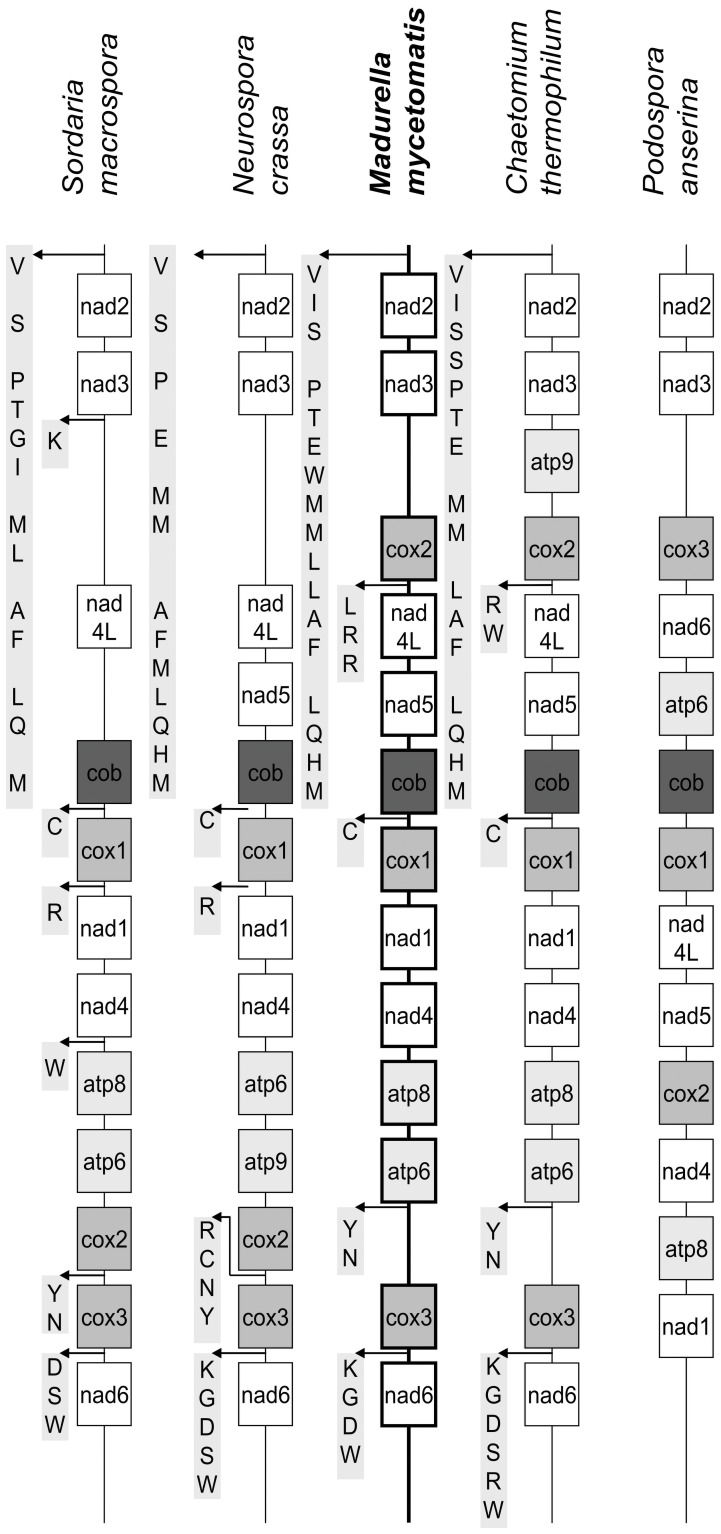
Mitochondrial gene order of 5 members of the order Sordariales. The gene order of the protein-encoding genes, *atp*6, *atp*8, *atp*9, *cob, cox*1, *cox*2, *cox*3, *nad*1, *nad*2, *nad*3, *nad*4, *nad*4L, *nad*5 and *nad*6 are shown for *S. macrospora, N. crassa, M. mycetomatis, C. thermophilum* and *P. anserina*. For the first four species, the positions of the tRNA genes are also depicted by using their one letter amino acid code.

The relatedness amongst the order Sordariales is further studied by comparing the mitochondrial organizatiopone.0038654.g002.tifn of *M. mycetomatis* to the 4 complete fungal mtDNA sequences belonging to the order Sordariales. Comparable to the high similarity in amino-acid sequence and the uniform mtDNA genome organization found for dermatophytes belonging to the order Onygenales [10], the mitochondrial genome organization found for the order Sordariales is apparently also uniform (figure 3). The only exception is the mitochondrial genome organization of *P. anserina,* which differs from the genome organization of the other members of the order Sordariales (figure 3). This marked difference has been noted in the past, and led to the conclusion that the mitochondrial gene order in the order Sordariales was apparently quite diverse [11]. Here it is shown, that for most mitochondrial genomes in the order Sordariales this is not the case. More mitochondrial genomes are needed for the order Sordariales to determine if the gene order is indeed similar and that *P. anserina* is the exception, or that the gene orders are in overall more diverse within this order. When comparing the different genome organizations it appears that the genome organization of *M. mycetomatis* is most closely related to that of *C. thermophilum* (figures 3), which only differed in the presence of the gene *atp*9 between *nad*3 and *cox*2 in *C. thermophilum* and its absence in *M. mycetomatis.* Next to having the same gene order, the tRNA clustering in the order Sordariales is similar. Again the tRNA order of *M. mycetomatis* resembles that of *C. thermophilum* the most (figure 3). Combining the phylogenetic data, the gene order and the tRNA order it appears that the mitochondrial genome of *M. mycetomatis* is most closely related to the mitochondrial genome of *C. thermophilum.* Fungi belonging to the order Sordariales are mostly soil-, wood- and dung-inhabiting fungi [43]. *N. crassa* is usually found in or on burned vegetation and the soil, while het natural habitat of *S. macrospora P. anserina* and *C. thermophilum*, is mainly the soil and herbivore dung [15], [44], [45], [46]. Although DNA of *M. mycetomatis* has been shown to be present in soil and on thorns in the endemic area, nobody has been able to culture *M. mycetomatis* directly from these niches [47]. Therefore the natural habitat of *M. mycetomatis* still needs to be confirmed. Based on this and other studies, it is demonstrated that *M. mycetomatis* clusters within the order Sordariales, therefore the natural habitat of *M. mycetomatis* might be sought on similar substrates. To discover the natural niche of this fungus could lead to strategies in the prevention of this mutilating disease.

### Conclusion

Comparative genomics provides a powerful tool for uncovering similarities and differences between species and placing them in their correct order. In order to gain insight in the evolutionary place of *M. mycetomatis* in the fungal kingdom, previous studies have used the nuclear ribosomal internal transcriped spacers (ITS) which showed that *M. mycetomatis* clusters amidst the order Sordariales. Here the complete mitochondrial genome of *M. mycetomatis* is reported. The composition and organization of the genes within this mtDNA are found to cluster amongst the Sordariales, and is found to be almost identical to that of *C. thermophilum.* Phylogenetic analyses of the whole protein-encoding gene content of *M. mycetomatis* confirm its position in the order of the Sordariales with *C. thermophilum* as its closest relative.
